# Proteomic analysis of differentially expressed proteins in hepatitis B virus-related hepatocellular carcinoma tissues

**DOI:** 10.1186/1756-9966-28-122

**Published:** 2009-08-28

**Authors:** Ning Li, Yunzhu Long, Xuegong Fan, Hongbo Liu, Cui Li, Lizhang Chen, Zhiming Wang

**Affiliations:** 1 Department of Blood transfusion, Xiangya Hospital, Central South University, Changsha 410008, PR China; 2 Department of Infectious Diseases, the First Hospital of Zhuzhou, Zhuzhou 412000, PR China; 3 Department of Infectious Diseases, Xiangya Hospital, Central South University, Changsha 410008, PR China; 4 Key Laboratory of Cancer Proteomics of Chinese Ministry of Health, Changsha 410008, PR China; 5 School of Public Health, Central South University, Changsha 410078, PR China; 6 Department of Surgery, Xiangya Hospital, Central South University, Changsha 410008, PR China

## Abstract

**Background:**

Hepatocellular carcinoma (HCC), a major cause of cancer death in China, is preceded by chronic hepatitis and liver cirrhosis (LC). Although hepatitis B virus (HBV) has been regarded as a clear etiology of human hepatocarcinogenesis, the mechanism is still needs to be further clarified. In this study, we used a proteomic approach to identify the differential expression protein profiles between HCC and the adjacent non-tumorous liver tissues.

**Methods:**

Eighteen cases of HBV-related HCC including 12 cases of LC-developed HCC and 6 cases of chronic hepatitis B (CHB)-developed HCC were analyzed by two-dimensional electrophoresis (2-DE) combined with matrix-assisted laser desorption/ionization time of flight mass spectrometry (MALDI-TOF-MS), and the results were compared to those of paired adjacent non-tumorous liver tissues.

**Results:**

A total of 17 differentially expressed proteins with diverse biological functions were identified. Among these, 10 proteins were up-regulated, whereas the other 7 proteins were down-regulated in cancerous tissues. Two proteins, c-Jun N-terminal kinase 2 and ADP/ATP carrier protein were found to be up-regulated only in CHB-developed HCC tissues. Insulin-like growth factor binding protein 2 and Rho-GTPase-activating protein 4 were down-regulated in LC-developed and CHB-developed HCC tissues, respectively. Although 11 out of these 17 proteins have been already described by previous studies, or are already known to be involved in hepatocarcinogenesis, this study revealed 6 new proteins differentially expressed in HBV-related HCC.

**Conclusion:**

These findings elucidate that there are common features between CHB-developed HCC and LC-developed HCC. The identified proteins are valuable for studying the hepatocarcinogenesis, and may be potential diagnostic markers or therapeutic targets for HBV-related HCC.

## Introduction

Hepatitis B virus (HBV)-related hepatocellular carcinoma (HCC) is one of the most common malignancy in the world, especially in China [1,2]. HCC is usually preceded by chronic hepatitis and liver cirrhosis (LC). The common clinical evolution from chronic hepatitis, LC and ultimately to HCC suggests that the carcinogenesis of HCC is a complex process involving multiple events and steps. Some molecular pathogenesis studies have been undertaken successfully on the gene (DNA) and transcription (mRNA) levels, however the carcinogenic mechanism of HBV-related HCC still remains poorly understood.

Development of high throughput proteomics approach provides a new tool to study the pathogenesis of HCC [3]. Proteomics has been used to characterize the molecular events occurring in various disease processes. The two-dimensional electrophoresis (2-DE) followed by mass spectrometry (MS) analysis is the principal step of proteomics to identify the comparative expression profiles at the protein level that may be associated with specific diseases. Such approaches are expected to establish the molecular definition of the nontumor and tumor states and contribute to the discovery of diagnostic markers and therapeutic targets.

There are already some previous proteomic studies for HCC, yet the proteomic analysis of HBV-related hepatocarcinogenesis still needs to be further clarified. The aim of the present study was to carry out a differential profiling of proteins from HBV-related HCC samples and their corresponding adjacent non-tumorous liver tissues including chronic hepatitis and LC tissue using matrix-assisted laser desorption/ionization time of flight mass spectrometry (MALDI-TOF-MS). The results presented here are expected to obtain some clues to further study the carcinogenic mechanisms, or identify some possible molecular markers for HBV-related HCC.

## Materials and methods

### Materials and chemicals

2-DE equipment, Imagescanner, ImageMaster 2D Elite 4.01 analysis software, semi-dry system (TE70 series Semi-Dry Transfer Unit), protein assay kit and supply materials (Immobiline DryStrips pH 3–10L, 24 cm, 13 cm, pharmalytes) were purchased from Amersham Biosciences. Other chemicals were mainly obtained from Amersham Biosciences. Trypsin was obtained from Sigma. All chemicals were of analytical reagent grade. Applied Biosystem Voyager -DETM STR Biospectrometry™ workstation System 4307 MALDI-TOF-MS was purchased from Applied Biosystems.

### Liver tissue samples

Human liver tissue samples used in this study were selected from 18 patients who had undergone partial hepatectomy for HBV-related HCC at the Xiangya Hospital during the period 2003 2005 [see Table 1]. All HCC patients were diagnosed based on clinical data, including image evidence, histopathological examination [4], and there was no evidence of co-infection with other hepatotropic viruses. Further possible causes of liver damage, such as alcohol, drugs or autoimmune diseases were also excluded. According to Edmonson pathologic grading, the18 cases are all grade I. Compared to the tumorous liver tissue, 18 nontumorous liver specimens (taken at a distance of at least 2 cm from the tumor) including 12 cirrhotic tissue (LC) samples and 6 chronic hepatitis B (CHB) tissue samples were also obtained from the same individuals respectively [5]. Both LC tissues and CHB tissues were diagnosed by pathological confirmation. The study was approved by the hospital ethnic committee, and all patients in the study were consentient before tissue donation.

**Table 1:** The main chracteristics of 18 HCC cases

Case	Gender	Age (years)	HBV infection				HCV	Histopathologic diagnosis of adjacent non-tumorous tissues
					
			HbsAg	HbeAg	anti-HBc	DNA	RNA	
1	M	48	+	+	+	+	-	liver cirrhosis
2	M	46	+	+	+	+	-	liver cirrhosis
3	F	72	+	+	+	+	-	liver cirrhosis
4	M	28	+	+	+	+	-	liver cirrhosis
5	M	55	+	+	+	+	-	chronic hepatitis
6	F	26	+	+		+	-	chronic hepatitis
7	M	58	+	+	+	+	-	liver cirrhosis
8	M	25	+	+	+	+	-	liver cirrhosis
9	M	41	+		+	+	-	liver cirrhosis
10	M	36	+	+	+	+	-	chronic hepatitis
11	M	48	+	+	+	+	-	liver cirrhosis
12	F	62	+	+	+	+	-	liver cirrhosis
13	M	39	+	+		+	-	chronic hepatitis
14	M	44	+	+	+	+	-	liver cirrhosis
15	M	50	+	+	+	+	-	liver cirrhosis
16	M	38	+	+	+	+	-	chronic hepatitis
17	F	41	+	+	+	+	-	chronic hepatitis
18	M	65	+		+	+	-	liver cirrhosis

The preparation of tissue protein samples was described previously by Li. et al.[6]. Briefly, representative fragments of tumorous and non-tumorous liver tissue were immediately used to extract the total proteins, or were snap-frozen in liquid nitrogen and stored at -80°C until used for liver protein preparation. The specimens were then carefully sampled, fixed in 10% formalin, embedded in paraffin and routinely processed for diagnosis purposes. A total of 30~80 mg tissues were grinded into powder in liquid nitrogen, dissolved in 400 μl lysis buffer consisting of 7 mol/L urea, 2 mol/L thiourea, 2% NP-40, 1% Triton X-100, 100 mmol/L DTT, 5 mmol/L PMSF, 4% CHAPS, 0.5 mmol/L EDTA, 40 mmol/L Tris, 2% pharmalyte, 1 mg/ml DNase I, and 0.25 mg/ml RNase A, then vortexed, incubated at room temperature for 2 hr. The mixture was centrifuged (15000 r/min, 30 min, 4°C). The supernatant was the total protein solution. The concentration of the total proteins was assayed with the protein assay kit (Amersham Biosciences) by comparison of the absorbance of the diluted mixtures to a standard curve of bovine serum albumin in the range of 0–50 μg/L.

### 2-DE and image analysis

2-DE was performed to separate proteins as described in our previous papers [6-8]. The first dimension isoelectric focusing (IEF) electrophoresis was performed using IPG gel strip (pH 3–10 NL, 24 cm) on IPGphor (Amersham Biosciences). Briefly, 400 μg of protein samples was diluted to 450 μL with a rehydration solution [7 mol/L urea, 2 mol/L thiourea, 0.2% DTT and 0.5% (v/v) pH 3–10 IPG buffer], and applied to IPG strips (pH 3–10L, 24 cm) by 14 h ehydration at 30 V. The proteins were focused successively for 1 h at 500 V, 1 h at 1,000 V, and 8.5 h at 8,000 V to give a total of 68 kVh on an IPGphor. Focused IPG strips were equilibrated for 15 min in a solution [6 mol/L urea, 2% SDS, 30% glycerol, 50 mmol/L Tris-HCl (pH 8.8), and 1% DTT], and then for an additional 15 min in the same solution except that DTT was replaced by 2.5% iodoacetamide. After equilibration, SDS-PAGE was done on Ettan DALT II system (Amersham Biosciences). After SDS-PAGE, gels were stained with silver nitrate according to the protocol of Plusone sliver staining kit (Amersham Biosciences). Each experiment was performed in triplicate.

2-DE maps were obtained by scanning the gels using the Imagescanner. Analysis of the gels was accomplished using the PDQuest analysis software including background subtraction, spots detection, volume normalization and the establishment of a reference gel. Intensity of each protein was quantified by calculation of spot volume after normalization of the image using the total spot volume normalization method multiplied by the total area of all the spots. The calculation of the theoretical molecular weight and pI values of the identified protein spots is based on algorithms included in the ImageMaster 2D Elite 4.01 analysis software package. Statistical analysis was carried out with SPSS for Windows 10.0 and Excel.

### MALDI-TOF-MS

Differential protein spots were excised from preparative gels using biopsy punches and transferred to a 1.5 ml siliconized Eppendorf tube. Proteins in-gel was digested as previously described [6]. The gel-spots were destained in the destaining solution consisted of 100 mmol/L Na_2_S_2_O_3 _and 30 mmol/L K_3_Fe(CN)_6 _(1:1). The proteins-contained gel-spots were reduced in the reduction buffer consisted of 100 mmol/L NH_4_HCO_3_, 10 mmol/L DTT for 1 h at 57°C, and alkylated in the alkylation buffer consisted of 100 mmol/L NH_4_HCO_3_and 55 mmol/L iodocetamide in the dark for 30 min at room temperature. The gel pieces were dried in a vacuum centrifuge. The dried gel-pieces were incubated in the digestion solution consisted of 40 mmol/L NH_4_HCO_3_, 9%ACN and 20 μg/mL trypsin(Sigma, St. Louis, USA) for 16 h at 37°C. The tryptic peptide mixture was extracted and purified with Millipore ZIPTIP™C18 column. The purified tryptic peptide mixture was mixed with α-cyano-4-hydroxycinnamic acid (CCA) matrix solution, and vortexed lightly. A volume (1 μl) of the mixture containing CCA matrix was loaded on a stainless steel plate, and dried in the air. The samples were analyzed with Applied Biosystems Voyager System 4307 MALDI-TOF Mass Spectrometer (ABI). The parameters were set up as following: positive ion-reflector mode, accelerating voltage 20 kV, grid voltage 64.5%, mirror voltage ratio 1.12, N_2 _laser wavelength 337 nm, pulse width 3 ns, the number of laser shots 50, acquisition mass range 1000–3000 Da, and delay 100 nsec, and vacuum degree 4×10^-7^Torr. A trypsin-fragment peak was served as internal standard for mass calibration. A list of the corrected mass peaks was the peptide mass fingerprinting (PMF).

### Database analysis

Proteins were identified with peptide mass fingerprinting data by searching software PeptIdent  and Mascot . Mascot Distiller was used to detect peaks by attempting to fit an ideal isotopic distribution to the experimental data. The searching parameters were set up as following[6,7]: the mass tolerance was ± 0.5 Dalton; the number of missed cleavage sites was allowed up to 1; the cysteine residue was modified as carbamidomethyl-cys; variable modifications was oxidation (M); the minimum number of matched-peptides was 5; species was selected as homo sapiens(Human); the peptide ion was [M+H]^+^; Mass values was monoisotopic; the searching range was within the experimental pI value ± 0.5 pH unit and experimental Mr ± 20%.

## Results

### 2-DE maps for human liver tissue proteome

**Figure 1:**
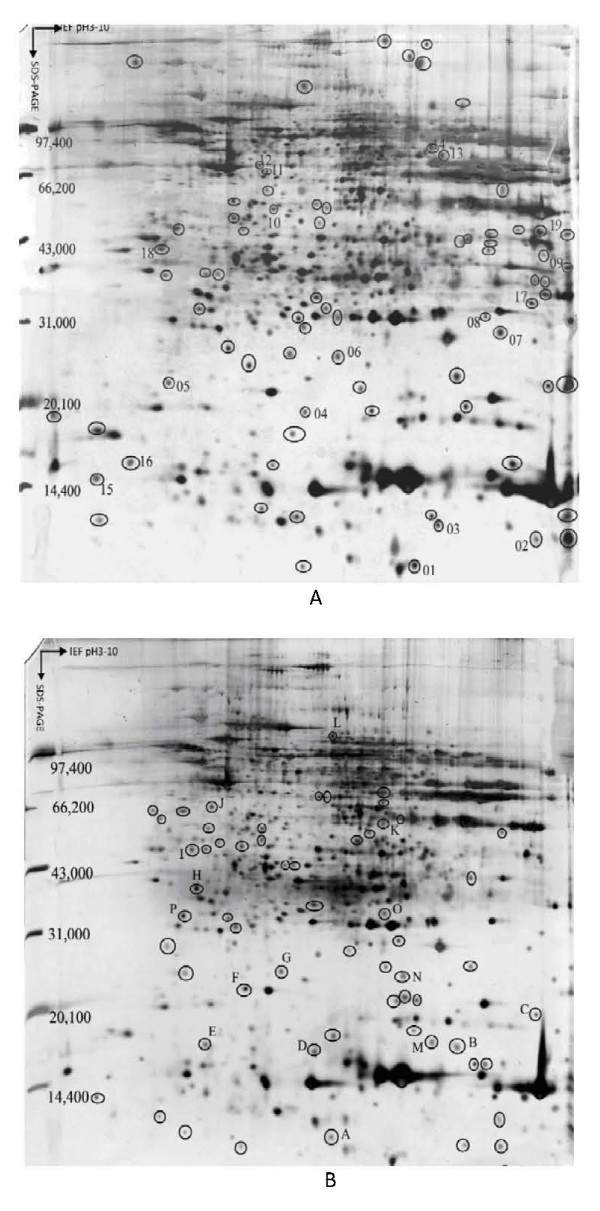
**Representative silver-stained 2-DE proteins maps obtained from (A) HCC tumorous tissue and (B) adjacent paired liver cirrhosis tissue**. The circled protein spots with Arabic numbers in (A) were up-regulated in tumorous tissues. The circled protein spots with English letters in (B) were up-regulated in cirrhotic tissues.

**Figure 2:**
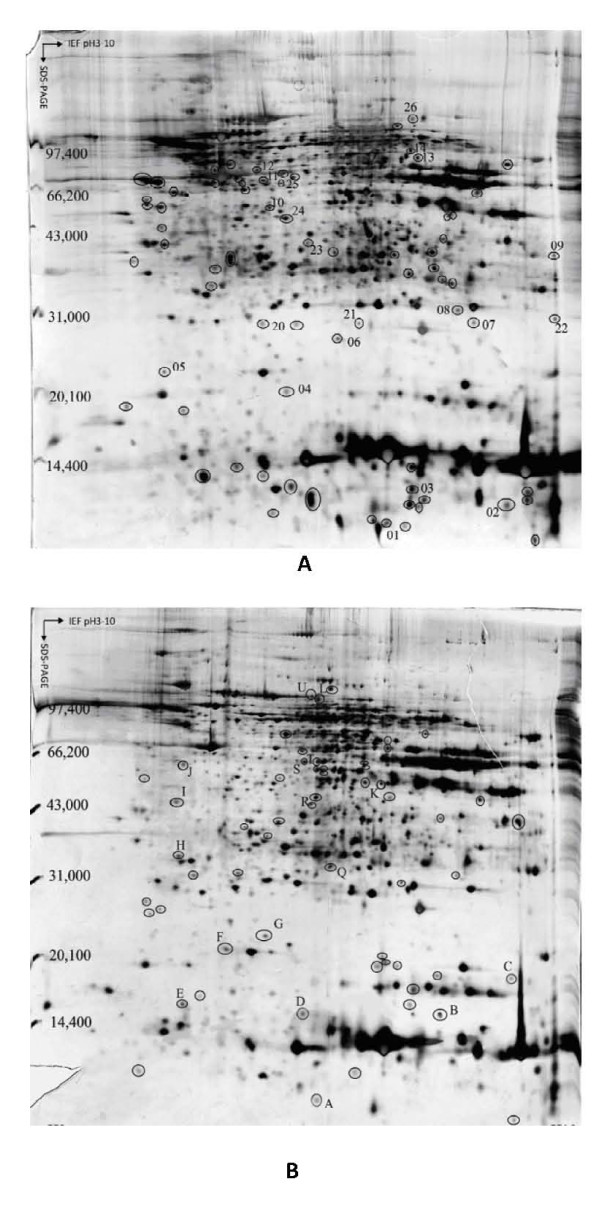
**Representative silver-stained 2-DE proteins maps obtained from (A) HCC tumorous tissue and (B) adjacent paired chronic hepatitis tissue**. The circled protein spots with Arabic numbers in (A) were up-regulated in tumorous tissues. The circled protein spots with English letters in (B) were up-regulated in chronic hepatitis tissues.

In order to validate the reproducibility, 2-DEs for 18 cases of HBV-related HCC including 12 cases of LC-developed HCC and 6 cases of CHB-developed HCC were repeated for three times. The image analysis showed that these 2-DE maps were reproducible. Using this technique, Over 1,000 protein spots were clearly separated on the gels, ranging from 1100–1400 massed between pH 3–10 in three different tissues. A total of 100 well-resolved and matched spots among three tumor-gels were chosen randomly to calculate the deviation of the spot position. The spot positional deviation was 2.47. ± 0.25 mm in the IEF direction, and 2.86 ± 0.25 mm in SDS-PAGE direction. For 12 cases of HCC developed from LC, a total of 1281 ± 51 spots were detected in tumorous tissues with an average matching rate of 94.38%, while a total of 1188 ± 41 spots were detected in LC tissues, with an average matching rate of 94.95%. For 6 cases of HCC developed from CHB, a total of 1245 ± 37 spots were detected in tumor tissues with an average matching rate of 94.69%, while a total of 1235 ± 31 spots were detected in hepatitis tissues with an average matching rate of 95.55%. The well-resolved and reproducible 2-DE patterns of HBV-related HCC tissues and non-tumorous liver tissues adjacent to tumors were attained, which are displayed in Figure 1 and Figure 2.

In this study, the 2-DE protein patterns of 12 pairs of tumor/cirrhosis samples and 6 pairs of tumor/hepatitis samples were quantified and mutually matched. In order to preselect protein variations, the protein patterns of tumor and nontumor tissues were set into two classes, and quantities of all detected spots in both classes were compared by the Student's t-test in ImageMaster 2-DE gel analysis software [6,8]. The 2-DE profiles were very similar among 18 tumor tissues samples. To construct a 2-DE map, it is important to have a representative sample. Hence, an average electrophoretic map of human HBV-related HCC tissues was constructed by the comparison of the 2-DE maps from 18 tumor tissues with the ImageMaster 2-DE gel analysis software. The average electrophoresis map included 2076 protein-spots. Similarly, two average electrophoresis maps of 12 cirrhotic tissues and 6 hepatitis tissues were also established with 1987 protein-spots and 1899 protein-spots, respectively. These average electrophoresis maps were used to perform the differential expression analysis. The Differential protein spots were defined as spots in 2-DE gels whose expression up-regulated significantly (at least three-fold discrepancy) in more than 50% certain tissue compared with control tissue. We compared the 2-DE protein patterns of the average gels of tumorous and cirrhotic tissue, 35 differential protein-spots were detected, among which 19 proteins were up-regulated in tumorous tissues, and 16 were up-regulated in cirrhotic tissues significantly. As shown in Figure 1, the spots numbers in part A stood for the proteins which were only expressed or over-expressed in tumorous tissues, and the spots numbers in part B stood for the proteins which were only expressed in cirrhotic tissues or down-regulated in tumorous tissues. We also compared the differential protein expression of paired tumorous and chronic hepatitis B liver tissues from 6 patients with HCC. We found that there were 38 differential spots between cancerous tissues and chronic hepatitis tissues, of which 21 differential protein spots were up-regulated in cancerous tissues, while 17 differential protein spots were up-regulated in chronic hepatitis tissues. As shown in Figure 2, the spots numbers in part A stood for the proteins which were only expressed or over-expressed in tumorous tissues, and the spots numbers in part B stood for the proteins which were only expressed in chronic hepatitis tissues or down-regulated in tumorous tissues.

### Identification of differentially expressed proteins in HCC developed from LC

**Figure 3:**
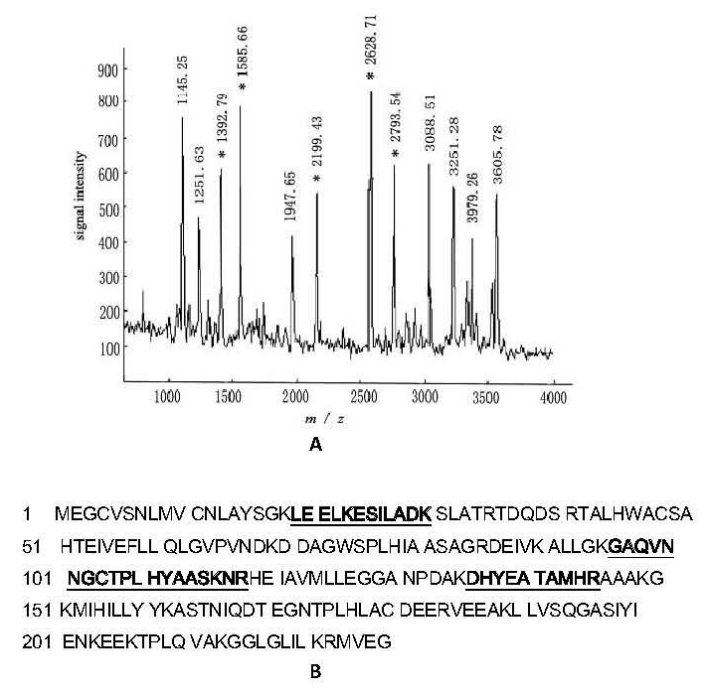
**MALDI-TOF-MS analysis of differential protein spot 6**. (A) The MALDI-TOF-MS mass spectrum of spot 6, identified as the Gankyrin according to the matched peaks is shown. (B) Protein sequence of Gankyrin is shown, and matched peptides are indicated in bold font and underlined.

The differential protein-spots were excised from the silver stained gels, and digested in-gel with trypsin. The peptide mass fingerprinting (PMF) maps were obtained by MALDI-TOF-MS, and calibrated with Trypsin auto-degraded peak (m/z = 1993.9772 Da). A selected PMF of protein spot 6 was display in Figure 3. The PMF data were used to search the SWISS-PROT, TrEMBL and NCBI databases with PeptIdent or Mascot software. The resulting protein was determined by comprehensively considering the corresponding experimental pI, Mr, the number of matched-peptides, and the sequence coverage. Among the 35 protein spots, PMF maps of 23 proteins were obtained by MALDI-TOF-MS, and 14 differential proteins were identified. Of the 14 identified proteins, the expressional levels of 8 proteins named Cytoplasmic dynein heavy chain, Nucleolar phosphoprotein B23, Gankyrin, Cytoskeletal 8, Alpha-1-fetoprotein, Lamin B1, Insulin-stimulated protein kinase 1 and Cell division cycle protein 27 homolog (CDC27Hs) were up-regulated in cancerous tissues [see Additional file 1], whereas the expressional levels of 6 proteins named Cyclin-dependent kinase inhibitor p12, Cyclin-dependent kinase inhibitor 1, Antioxidant protein 2, Protein disulfide isomerase A2, C-1-tetrahydrofolate synthase and Insulin-like growth factor binding protein 2 were up-regulated in cirrhotic tissues [see Additional file 1].

### Identification of differentially expressed proteins in HCC developed from CHB

Thirty eight differential spots between cancerous tissues and chronic hepatitis tissues had been observed. Using MALDI-TOF-MS, 24 PMF were successfully obtained, and 16 proteins were identified. Among the 16 identified proteins, 10 proteins were found to be up-regulated in HCC developed from CHB. The up-regulated 10 proteins included 8 above described proteins over-expressed in HCC developed from LC and other two proteins named c-Jun N-terminal kinase 2 and ADP/ATP carrier protein [see Additional file 1]. Six proteins out of 16 identified proteins including 5 above-mentioned proteins which were up-regulated in cirrhotic tissues (Cyclin-dependent kinase inhibitor p12, Cyclin-dependent kinase inhibitor 1, Antioxidant protein 2, Protein disulfide isomerase A2, C1-tetrahydrofolate synthase) and Rho-GTPase-activating protein 4 were up-regulated in chronic hepatitis tissues [see Additional file 1].

## Discussion

HCC is one of the most fatal cancers worldwide, and it is responsible for approximately one million deaths each year. Though the HBV infection is regarded as the most clearly established risk factors, the mechanism is complex and the distinct molecular pathway or molecules involved this phenomenon still remains poorly understood. The possible carcinogenic mechanism of HBV-related HCC is related to the long term-inflammatory changes caused by HBV infection. Chronic hepatitis and cirrhosis are two phases of hepatic necrotizing inflammation caused by HBV infection. Each year, approximate 2%~3% patients with LC will develop HCC, and 0.2% patients with CHB will develop HCC [9,10]. Few studies have been reported concerning the difference between LC-developed HCC and CHB-developed HCC.

MALDI-TOF-MS is a new technique identifying proteins. Since it can rapidly provide a protein expression profile from a variety of biological and clinical samples, many tumorous tissues proteomic studies have been carried out by using this system [11-13]. In this study, the comparative proteomic study was performed between the HCC tissues and the adjacent no-tumorous tissues including CHB and LC tissues. Seventeen differential protein-spots were identified by MALDI-TOF-MS-based PMF analysis. Eight out of 17 proteins were found to be up-regulated in tumorous tissues of HCC developed from CHB as well as developed from LC. These included Cytoplasmic dynein heavy chain, Nucleolar phosphoprotein B23, Gankyrin, Cytoskeletal 8, Alpha-1-fetoprotein, Lamin B1, Insulin-stimulated protein kinase 1 and Protein CDC27HS. Meanwhile, five out of 17 proteins, named Cyclin-dependent kinase inhibitor p12, Cyclin-dependent kinase inhibitor 1, Antioxidant protein 2, Protein disulfide isomerase A2, C1-tetrahydrofolate synthase were down-regulated both in LC-developed HCC and CHB-developed HCC. However, two identified proteins, c-Jun N-terminal kinase 2 and ADP/ATP carrier protein were found to be up-regulated only in CHB-developed HCC tumorous tissues. The expressions of insulin-like growth factor binding protein 2 and Rho-GTPase-activating protein 4 were up-regulated in LC liver tissues and CHB liver tissues, respectively. Classification of all proteins [see Additional file 1] showed that HCC is such a complicated disease involving multiple-aspects and genes in the differentially expressed proteome at the level of whole-cell extract. Although a few special proteins differentially expressed in CHB-developed HCC or LC-developed HCC, most of identified proteins expressed in both CHB-developed HCC and LC-developed HCC, which indicates that there are common features between CHB-developed HCC and LC-developed HCC.

Among the 17 proteins identified in this study, 11 proteins have been already described by previous studies, or are already known to be involved in hepatocarcinogenesis. These proteins are involved in cell growth, proliferation, differentiation, metabolism, cell cycle regulation, cytoskeleton and signal transduction. Importantly, 6 novel proteins including 3 up-regulated proteins (CDC27Hs, ADP/ATP carrier protein, Insulin-stimulated protein kinase 1) and 3 down-regulated proteins (Rho-GTPase-activating protein 4, Antioxidant protein 2, C1-tetrahydrofolate synthase), were identified in our study. Although these proteins were obtained on a limited number of patients, it should be pointed out that our analysis correctly identified the vast majority of the proteins previously known to be regulated in HCC. It is thus reasonable to assume that the newly identified proteins may be involved in the development of hepatocarcinogenesis or are potential markers of HCC. As a cell cycle regulator, CDC27Hs colocalizes to the centrosome at all stages of the mammalian cell cycle, and to the mitotic spindle. Injection of affinity-purified anti-CDC27Hs antibodies into logarithmically growing HeLa cells caused a highly reproducible cell cycle arrest in metaphase with apparently normal spindle structure [14]. Some studies indicated that CDC27Hs may be involved in the cancer cell growth [15,16]. The role of CDC27Hs in hepatocarcinogenesis needs further study. ADP/ATP carrier protein (AAC) was found to be up-regulated in a larger series of HCC tissues in this study, but down-regulated in notumorous tissues especially in chronic hepatitis B tissues. AAC is an integral protein present in the inner mitochondrial membrane, which performs the exchange of cytoplasmic and intramitochondrial ADP and ATP. Schulze *et al *considered the dysfunction of ACC can be induced by some viral infection such as coxsackie B3 virus and leads to an imbalance in myocardial energy metabolism, which is responsible for the impairment of cardiac function [17,18]. Recently, the over expression of AAC has already been observed in breast cancer cell [19], and AAC was regarded as a potential biomarker for therapy and prognosis in breast cancer.

The 3 novel down-regulated proteins in this study are mainly involved in metabolism, oxidative stress and proliferation. Rho-GTPase-activating protein 4 (ARHGAP4) is a member of the Rho GTPase activating protein (RhoGAP) family. The RhoGAP family proteins play an important role in regulating cell migration, cell morphology and cytoskeletal or ganization [20]. The RhoGAP transcripts were found to be truncated or lowly expressed in some breast carcinoma cell lines, indicating that loss of RhoGAP or its altered activity may suppresse the growth of breast tumor cells [21]. Deleted in liver cancer-1 gene (DLC-1) which is isolated from human hepatocellular carcinoma and encodes a Rho GTPase-activating protein, is frequently inactivated or down-regulated in liver and prostate carcinoma cells [22]. As a tumor suppressor gene, DLC1 significantly inhibits cell proliferation, reduced the motility and invasiveness of hepatocellular carcinoma cells [23]. Our results in this study showed a low expression of ARHGAP4 at the protein level in 83% of 6 human HCC tested [see Additional file 1]. However, no data have been given to demonstrate the role of ARHGAP4 in hepatocarcinogenesis till now, and the relationship between ARHGAP4 and DLC1 need to be further evaluated. Antioxidant protein 2(AOP2), a unique member of the thiol-specific antioxidant family of proteins, has been shown to remove H_2_O_2 _and protect proteins and DNA from oxidative stress [24,25]. Oxidative damage usually leads to decrease ATP level and consequently play an important role in carcinogenesis and metastasis of HCC [26,27]. Increased expression of the stress proteins such as HSP, heat shock cognate (HSC), glucose-regulated protein (GRP) and glycolytic enzymes was found in HCC using 2-DE-based proteomics [28]. Ezzikouri et al further defined that hepatitis B and C viruses may induce chronic inflammation and oxidative stress, which could predispose a cell to mutagenesis and proliferation [29]. Decreased expression of AOP2 has been previously reported in human prostate cancer [30] and colon cancer cells [31]. In this study, AOP2 was firstly found to be down-regulated in HCC tissues, indicating that HCC cells are in a state of elevated stress and stimulated metabolism. C(1)-tetrahydrofolate (THF) synthase, the eukaryotic trifunctional enzyme, interconvert folic acid derivatives between various oxidation states and is critical for normal cellular function, growth, and differentiation [32]. Howard et al found that the expression patterns of C(1)-THF synthase was involved in liver regeneration [33]. The function and acting mechanisms of this protein await further study.

In conclusion, using 2-DE combined with MS and a stringent statistical analysis, we have been able to identify the differentially proteomic analysis of human HBV-related HCC developed from CHB or LC. Several identified proteins may be potential tumor markers or promising new candidate actors for liver carcinogenesis. Functional studies on selected targets are underway to confirm this hypothesis.

## Conflict of interest statement

The authors declare that they have no competing interests.

## Authors' contributions

NL carried out the 2-DE, participated in MALDI-TOF-MS and drafted the manuscript. YL participated in MALDI-TOF-MS and performed the database analysis. XF is the corresponding author, conceived of the study and designed the study. HL participated in the preparation of tissue protein. CL mainly participated in the database analysis. LC participated in the design of the study and coordination. ZW participated in the collection of liver tissue samples. All authors read and approved the final manuscript.

## Supplementary Material

Additional file 1 **Identified proteins in HCC tissues using MALDI-TOF-MS**. The data provided 17 identified proteins in HCC tissues including 10 up-regulated proteins and 7 down-regulated proteins. Click here for file
