# Supplementary material for: Proteomic analysis of differentially expressed proteins in hepatitis B virus-related hepatocellular carcinoma tissues
Source: J Exp Clin Cancer Res. 2009 Aug 28;28(1):122. doi: 10.1186/1756-9966-28-122 (PMC2743659; doi:10.1186/1756-9966-28-122)
Supplement: Additional file 1 — Identified proteins in HCC tissues using MALDI-TOF-MS. The data provided 17 identified proteins in HCC tissues including 10 up-regulated proteins and 7 down-regulated proteins. [file 1756-9966-28-122-S1.doc]

Identified proteins in HCC tissues using MALDI-TOF-MS

Spot Accession pI Mass weigh Score Peptides Ratio of intensity Protein Frequency ^h)^ Protein name Functional

No ^a)^ ID ^b)^ (Da) matched A ^f)^ B ^g)^ coverage 1 ^i)^ 2 ^j)^ classification

**Ten up-regulated proteins in tumor tissues**

04 O00432 6.32 19,661 82 5/13 4.9 4.1 25.2％ 50％ Cytoplasmic dynein heavy chain Signal transduction

05 Q9BTI9 4.82 23,547 94 7/11 7.7 6.5 49.1％ 72.2％ Nucleolar phosphoprotein B23 Cell growth

06 O75832 6.73 24,428 87 5/12 10.9 6.3 37.6％ 100％ Gankyrin Cell cycle regulation

10 P05787 5.61 53,543 69 5/13 4.9 4.4 32.7％ 83.3％ Cytoskeletal 8 Structure

11 P20700 5.54 66,277 67 5/16 3.3 8.1 34.6％ 100％ Lamin B1 Cell proliferation

12 P02771 5.52 68,677 85 6/17 5.4 5.9 39.5％ 72.2％ Alpha-1-fetoprotein Metabolism

13 P51812 7.55 83,736 75 7/20 4.1 4.3 24.9％ 100％ Insulin-stimulated protein kinase 1 Signal transduction

14 P30260 7.24 91,867 81 5/15 10.2 8.0 30.3％ 55.6％ CDC27Hs Cell cycle regulation

22 ^c)^ P12235 9.64 33,064 79 7/16 1.9 24.3 28.7％ 100％ ADP/ATP carrier protein Cell growth

24^c)^　P45984 6.25 48,112 83 6/14 1.1 5.9 36.6％ 100％ c-Jun N-terminal kinase 2 Signal transduction

**Seven down-regulated proteins in tumor tissues**

A O95440 6.82 12,212 86 5/9 3.6 4.2 42.7％ 83.3％ 100％ Cyclin-dependent kinase inhibitor p12 Cell cycle regulation

C P38936 9.24 18,119 72 5/13 4.0 4.9 28.3％ 91.7％ 83.3％ Cyclin-dependent kinase inhibitor 1 Cell cycle regulation

G P30041 6.28 24,904 83 7/19 6.7 5.3 40.9％ 100 ％ 83.3％ Antioxidant protein 2 Antioxidant

J Q13087 5.04 58,206 77 6/18 4.3 3.7 31.5％ 50％ 66.7％ Protein disulfide isomerase A2 Metabolism

L  P11586 6.79 101,428 73 7/23 4.2 3.8 25.6％ 100％ 100％ C-1-tetrahydrofolate synthase Metabolism

O^d)^ P18065 7.31 35,137 92 7/14 6.3 1.3 42.1％ 50％ 50％ Insulin-like growth factor binding protein 2 Cell proliferation

U^e)^  P98171 6.73 105,055 79 9/22 1.5 4.3 25.5％ 91.7％ 83.3％ Rho-GTPase-activating protein 4 Cell proliferation

1. According with the numbers in Fig. 1 and Fig.2.
2. Swiss-Prot accession number.
3. Up-regulated protein found only in HCC developed from chronic hepatitis B.
4. Down--regulated protein found only in HCC developed from liver cirrhosis.
5. Down--regulated protein found only in HCC developed from chronic hepatitis B.
6. Ten up-regulated proteins in tumor tissues: intensity of cancerous tissues/intensity of cirrhotic tissues; Seven down-regulated proteins in tumor tissues: intensity of cirrhotic tissues / intensity of cancerous tissues.
7. Ten up-regulated proteins in tumor tissues: intensity of cancerous tissues/intensity of chronic hepatitis tissues; Seven down-regulated proteins in tumor tissues: intensity of chronic hepatitis tissues / intensity of cancerous tissues.
8. Frequency in 10 up-regulated proteins in tumor tissues means the proteins expression frequency in 18 cancerous tissues samples.
9. Frequency in 7 down-regulated proteins in tumor tissues means the proteins expression frequency in 12 liver cirrhosis tissues samples.
10. Frequency in 7 down-regulated proteins in tumor tissues means the proteins expression frequency in 6 chronic hepatitis tissues samples.
